# Multisensory integration augmenting motor processes among older adults

**DOI:** 10.3389/fnagi.2023.1293479

**Published:** 2023-12-20

**Authors:** Zhi Zou, Benxuan Zhao, Kin-hung Ting, Clive Wong, Xiaohui Hou, Chetwyn C. H. Chan

**Affiliations:** ^1^Department of Sport and Health, Guangzhou Sport University, Guangzhou, China; ^2^University Research Facility in Behavioral and Systems Neuroscience, The Hong Kong Polytechnic University, Hong Kong, Hong Kong SAR, China; ^3^Department of Psychology, The Education University of Hong Kong, New Territories, Hong Kong SAR, China

**Keywords:** multisensory integration, motor response, aging, lateralized response potential, event-related potential

## Abstract

**Objective:**

Multisensory integration enhances sensory processing in older adults. This study aimed to investigate how the sensory enhancement would modulate the motor related process in healthy older adults.

**Method:**

Thirty-one older adults (12 males, mean age 67.7 years) and 29 younger adults as controls (16 males, mean age 24.9 years) participated in this study. Participants were asked to discriminate spatial information embedded in the unisensory (visual or audial) and multisensory (audiovisual) conditions. The responses made by the movements of the left and right wrists corresponding to the spatial information were registered with specially designed pads. The electroencephalogram (EEG) marker was the event-related super-additive P2 in the frontal-central region, the stimulus-locked lateralized readiness potentials (s-LRP) and response-locked lateralized readiness potentials (r-LRP).

**Results:**

Older participants showed significantly faster and more accurate responses than controls in the multisensory condition than in the unisensory conditions. Both groups had significantly less negative-going s-LRP amplitudes elicited at the central sites in the between-condition contrasts. However, only the older group showed significantly less negative-going, centrally distributed r-LRP amplitudes. More importantly, only the r-LRP amplitude in the audiovisual condition significantly predicted behavioral performance.

**Conclusion:**

Audiovisual integration enhances reaction time, which associates with modulated motor related processes among the older participants. The super-additive effects modulate both the motor preparation and generation processes. Interestingly, only the modulated motor generation process contributes to faster reaction time. As such effects were observed in older but not younger participants, multisensory integration likely augments motor functions in those with age-related neurodegeneration.

## 1 Introduction

Adaptive behaviors rely on successful extraction of information from both the physical and social environments. The information is likely to be multimodal in nature while individuals encode, recognize, and integrate them (such as a verbal greeting from a person with a familiar face) before an appropriate response (such as nodding the head) is to be generated (Van Wanrooij et al., 2009; Magnotti and Beauchamp, 2017). Congruent multimodal stimuli such as audiovisual information was found to promote faster and more accurate responses than receiving audial or visual stimuli (Van Wanrooij et al., 2009; Tang et al., 2016). Luan et al. (2021) explained that faster motor sequence responses were due to the enhanced contiguous action effects (Luan et al., 2021). Other studies suggested that the response augmentation was due to a super-additive effect occurred when congruent multimodal stimuli are processed (Ren et al., 2017; Zou et al., 2017).

Neurodegeneration results in a general decline in both the sensory-encoding and motor-response processes (Mahoney et al., 2011; Wiegand et al., 2013; Amenedo et al., 2014; Zanto and Gazzaley, 2014). Declines were proposed to be due to lowered age-related sensory processing (Toledo et al., 2016), attention function (Zanto and Gazzaley, 2014), and response selection (Thoenes et al., 2018) resulting in prolonged motor execution (Wiegand et al., 2013; Amenedo et al., 2014). Our prior work indicated that the participants showed faster reaction times in the congruent multisensory than unisensory conditions (Zou et al., 2017). Between the older and younger participants, only the older adults showed significant associations between the faster reaction times and a supper-additive effect (Zou et al., 2017). The super-additive effect was the event-related potential (ERP) frontal P2 component elicited at 200 ms after stimulus onset. However, it is not clear how the super-additive effects, a cognitive component, relates to the faster reaction time, a motor executive function. The current study was aimed to investigate the plausible motor related processes involved in the faster reaction time by the audiovisual integration effect.

Major neural processes associated with motor responses in a choice-reaction task are stimulus detection and discrimination, motor preparation, and motor generation (Miller and Low, 2001). For sensory inputs, Cappe et al. (2010) reported enhanced early visual perception (less-positive C1) and stimulus evaluation and feedback (less-positive P2) in the multisensory condition (Stekelenburg and Vroomen, 2007; Cappe et al., 2010; Wang and Chan, 2015). For motor outputs, there are two processes. Firstly, as in the choice-reaction task, the choice process of responses can be reflected by the stimulus-locked lateralized readiness potential (s-LRP) (Slobounov, 2010). Secondly, the generation process of responses can be reflected by the response-locked readiness potential (r-LRP) (Yordanova et al., 2004; Luck and Kappenman, 2011). Both s-LRP and r-LRP are negative potentials observed over the sensorimotor areas contralateral to the hand eliciting the response (Coles, 1989). Their amplitudes are the magnitudes of neural activities accumulated over the contralateral motor cortex relative to the ipsilateral cortex for producing the motor process (van Vugt et al., 2014). The s-LRP references to the stimulus onset, while the r-LRP references to the response onset. The amplitude and onset latency of the s-LRP can reflect the motor preparation process and its speed, while those of the r-LRP reflect the motor generation process (Masaki et al., 2004). This study employed the s-LRP and r-LRP as the markers to reflect the motor preparation (or choice selection) and generation processes associated with the audiovisual integration. Multisensory conditions were found to result in shorter s-LRP latency than the audial or visual condition (Jepma et al., 2009). All the studies cited above, however, were based on younger but not older adults.

A handful of studies have been found on older individual’s LRPs of older adults. For instance, older adults were revealed to have more delayed s-LRP and r-LRP latencies than their younger counterparts (Wild-Wall et al., 2008; Cid-Fernández et al., 2014; Brush et al., 2020). The slow motor responses of the older adults were reported to attribute to the response generation process measured by the LRPs (Wiegand et al., 2013). Brush et al. (2020) later revealed that age-related slowing was related to decreases in both the s-LRP and r-LRP (Brush et al., 2020). Taken together, this study had two aims. Firstly, we investigated the neural processes possibly involved in the audiovisual integration enhanced motor responses. Secondly, the aging effects on modulating the motor related processes were explored. The multisensory integration effect was elicited with the audiovisual task previously described in Zou et al. (2017). The super-additivity ERP marker was the fronto-central P2. The motor related processes were the s-LRP and r-LRP. We hypothesized that, when compared with the single modality conditions, audiovisual condition would shorten the reaction time for both younger and older participants. The audiovisual condition would modulate both motor preparation and generation processes. These processes would be associated with faster reaction time for the older but not the younger participants. The results enable better understanding on the cognitive-to-motor effects brought by audiovisual integration in older adults.

## 2 Materials and methods

### 2.1 Participants

There were 29 younger (16 males, mean age: 24.9 years) and 31 older adults (12 males, mean age: 67.7 years) recruited in this study. The exclusion criteria were as follows: (1) received long-term musical training experience (>1 year) which may have confounded experimental task tapping on audiovisual integration (Zatorre et al., 2007); (2) diagnosed with neurological or muscular diseases or auditory impairment; (3) general cognitive decline assessed by Montreal Cognitive Assessment (MoCA) score of 22 or below. All participants passed the standard logarithmic visual acuity chart test (>0.8) and were able to differentiate the different “Bat-ears” sounds which were the auditory stimuli used in the present study. Ethics approval was obtained from the Departmental Research Committee of the institution which the first author was affiliated with (No.: 20140627001).

### 2.2 Stimuli

The stimuli used in the audiovisual motor response task were the same as those described in Zou et al. (2017) study. They were the visual and audial stimuli.

#### 2.2.1 Visual stimuli

**Figure 1:**
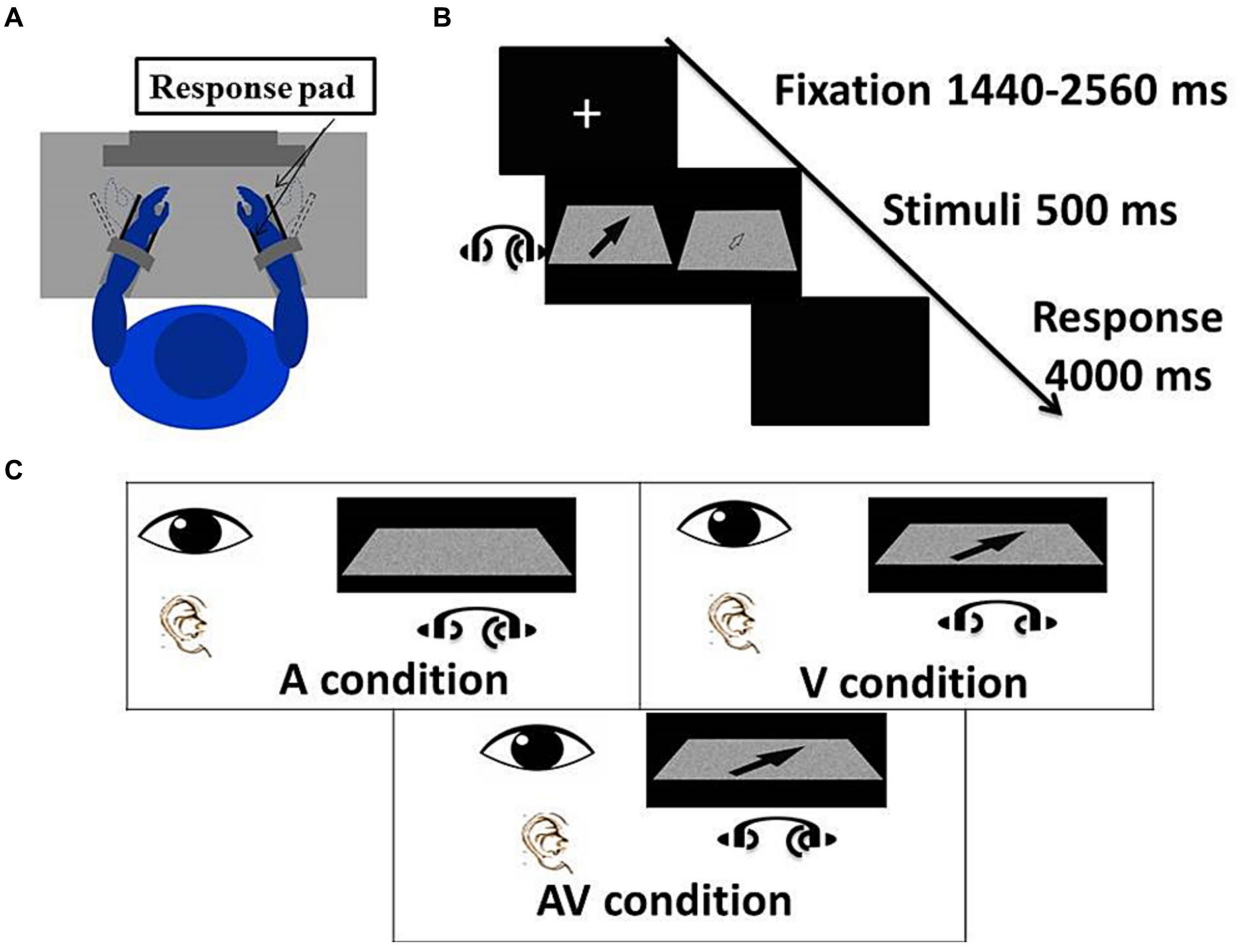
Experimental procedure and conditions. **(A)** The participant sat comfortably in front of a screen 80 cm from the eyes. Each hand was placed between the two response keys. **(B)** The experimental task. In each trial, a fixation was first presented at the center of the screen with a random duration from 1,440 to 2,560 ms. Visual and auditory stimuli were presented for 500 ms, which either one or both stimuli was indicative of a direction. For the sake of reading, the left arrow was totally clear, and the right arrow showed the actual visual stimuli, which was blurred to different levels based on the accuracy rate at subject level. A blank screen was presented for 4,000 ms and participants had to indicate the direction of visual and/or auditory stimuli. **(C)** There were three conditions in the experiment with different combinations of visual and auditory stimuli. In the A condition, lateralized “Bat-ears” sound and pure visual noise were presented. In the V condition, visual noise with an additional arrow pointing to one of the 4 directions and non-lateralized “Bat-ears” sound were presented simultaneously. The AV condition was composed of a visual noise with an arrow and lateralized “Bat-ears” sound. This figure is adapted from another paper from our lab (Zou et al., 2017).

The design was an arrow in a 3D space. The background of the space was a Gaussian visual-noise board to create a blurriness effect. The head of the arrow pointed to one of four directions: left-far (45°), right-far (135°), left-near (315°), and right-near (225°). All arrows were presented within the participant’s foveal region with an internal edge of 0.7°, external edge of 1.7°, and the center point of 1.2° in the visual field. This was to ensure participants had a clear view of the visual stimuli as well as prevent unnecessary eye movements during the experiment (Bargh and Chartrand, 2000) (Figure 1B). To increase participants’ effort for encoding the arrows, Photoshop software (version CS3 10.0; Adobe Systems, San Jose, CA) was used to add blurring effects to images of the arrows (0 for total blur and 100 for total clear). There were 40 visual stimuli, 10 in each of the four directions. The visual control stimuli were made of the Gaussian visual-noise boards presented in the auditory-only condition. Calibration of the visual stimuli showed 75–90% accuracy rates corresponding with blurriness levels of 29–60 (mean = 37) for the younger participants, and 40–90 (mean = 76) for the older participants (Zou et al., 2017).

#### 2.2.2 Auditory stimuli

The audial stimuli were adopted from the electronic “Bat-ears” device (Chan et al., 2012). The stimuli were echoes recorded from the ultrasound signals rebounded from obstacles placed at various locations in an acoustic laboratory. The echoes were “da-da-da” sounds embedded with spatial information representing near (1 m) and far (4 m) distances, and directions in terms of azimuth of ±30°. These gave four categories of sounds: left-far (azimuth 45°, 4 m), right-far (azimuth 135°, 4 m), left-near (azimuth 315°, 1 m), and right-near (azimuth 225°, 1 m). The sounds generated from the obstacle located in the middle position (azimuth of 0°, 1 m) served as the controls presented in the visual-only condition. There were 40 auditory stimuli, 10 in each of the four direction categories. All auditory signals were delivered via an earphone. The pitch and intensity of the sounds were within 2,600–4,900 Hz and 30–55 dB (Tao, 2015).

### 2.3 Audiovisual spatial discrimination task

The three experimental conditions were the visual [V], auditory [A], and audiovisual [AV] conditions. In the [V] condition, the visual stimulus was presented simultaneously with a control sound. The participant was asked to respond solely based on the visuospatial information encoded from the visual stimulus. In the [A] condition, the audial stimulus was presented simultaneously with a control visual Gaussian noise board. The participants were asked to respond based on the spatial information encoded from the audial stimulus. In the [AV] condition, the visual and audial stimuli were presented simultaneously, and the participant was asked to respond accordingly. The spatial information conveyed in the visual and audial stimuli in the [AV] condition was always congruent (Figure 1C).

A task trial began with a white fixation cross mounted on a black screen for 1,440–2,560 ms (mean 2000 ms) in randomized orders. The visual, audial, or audiovisual stimulus was presented for 500 ms after which the participant was to indicate the location presented by the stimulus by hitting a response pad with extension or flexion of the right or left wrist as accurately and as quickly as possible (Figure 1B). Movement responses of the wrist depended on the locations encoded from the stimuli. Left or right wrist indicated the left or right direction, while extension or flexion movement in the wrist indicated the far or near distance. The duration available for participants to respond was 4,000 ms. The inter-trial interval (ITI) ranged between 5,740–6,660 ms (mean 6,200 ms). A total of 672 trials were divided into eight blocks, with each block containing [A], [V], and [AV] conditions. The sequence of the three conditions within one block was in random order. The task took approximately 3.5 h to complete including rest periods (Table 1).

**Table 1:** Accuracy rate of performing audiovisual integration tasks in [A], [V], and [AV] conditions in both younger and older groups.

Accuracy rate	[A] condition	[V] condition	[AV] condition
Younger	0.80 ± 0.07	0.79 ± 0.07	0.92 ± 0.05
Older	0.66 ± 0.11	0.73 ± 0.12	0.85 ± 0.10

Data was presented as mean ± standard deviation. A, audial; V, visual; AV, audiovisual.

### 2.4 Procedures

The procedures were similar to those reported in the paper (Zou et al., 2017). The participants sat comfortably in a chair with the monitor 80 cm from the eyes. The forearms of the participants were strapped in the neutral position on an elbow-height table with the elbow flexed at 90° and the wrist at 0° with shoulder internal rotation. Two response pads (5 cm × 3 cm) were placed parallel on both sides of each hand and the participant needed to indicate the direction of the stimuli by wrist flexion or extension by approximately 30°. The distance between the two pads could be adjusted to fit the thickness of palm for each participant (Figure 1A). Both response type and time were recorded by the response pads via a computer. The three target stimuli conditions were presented randomly. Before each block commencement, the participants were informed of the task process and were required to respond as accurately and quickly as possible. As soon as they responded, the participants were required to reset their wrist to the neutral position.

### 2.5 Data acquisition

ERP data were captured with a 64-channel NuAmps Digital DC EEG Amplifier (Compumedics Neuroscan, USA Ltd). The sampling rate was set at 1024 Hz. The ground electrode was placed on the forehead and the montage reference was set at the right mastoid process. Reference impedance was <5 kΩ and inter-electrode impedance was <10 kΩ. A digital band-pass filter (0.1–30 Hz) was used (Zou et al., 2017). Only trials with correct responses were further analyzed. Epochs for analyzes were extracted between −200 ms and 2000 ms from stimulus onset. Epochs were discarded when amplitudes exceeded 100 μV. A covariance analysis algorithm was used to correct EEG data when the associated vertical and horizontal electrooculorams (EOGs) exceeded ±100 μV.

### 2.6 Data analysis

Detailed analyzes of the behavioral data and ERP data will not be described here as they were presented previously (Zou et al., 2017).

For the LRP signals, time window between −200 to 0 ms of the stimulus onset was set as the baseline for both the s-LRP and r-LRP. The epoched data were averaged in each condition for the C3 and C4 electrodes. After averaging, LRPs were calculated based on the formula LRP = (mean [C4-C3]^left hand^ + mean [C3-C4]^right hand^)/2 (Coles, 1989). Onset latency of LRPs was defined as the time when the amplitude began to rise and exceed 50% of the peak amplitude (Miller et al., 1998; Ulrich and Miller, 2001). Between-condition comparisons of LRP onset latency were conducted using the jackknife method which tackles the relatively large within-group variability (Wild-Wall et al., 2008; Cespon et al., 2013). As the s-LRP is a stimulus-locked component occurred around 800 ms after the stimulus, its time-window was 700 to 1,500 ms post-stimulus, while the r-LRP is a response-locked component and occurred at the instance when response is made, its time window was set as −200 to 200 ms post-response (Kolev et al., 2006; Frame, 2014). Differences in mean amplitudes of the s-LRP and r-LRP were tested between the unisensory ([A] or [V]) and multisensory ([A + V]) conditions in each of the younger and older groups. Two-way repeated analyzes of variance (ANOVAs) were used to test condition ([A], [V] and [A + V]) and age (younger and older) as well as their interaction effect (condition × age) on the mean amplitudes of the s-LRP and r-LRP. In the post-hoc analysis, paired-t test was used to test the between-condition differences. The statistical significance level was *p* ≤ 0.025 after Bonferroni correction for the two dependent variable comparisons.

Pearson correlation analysis was conducted between the behavioral data (IES) and the amplitude of both the s-LRP and r-LRP to assess the relationship between the motor process and behavioral performance.

## 3 Results

### 3.1 Behavioral results

As reported previously (Zou et al., 2017), the IES in both younger and older groups improved significantly in the [AV] condition compared with the unisensory ([A] or [V]) conditions. However, older participants had a larger difference in IES between [AV] and [V] conditions as well as between [AV] and [A] conditions compared to their younger counterparts. The results indicated that older adults received more behavioral performance benefit from multisensory integration.

### 3.2 ERP results

As reported in a previous paper (Zou et al., 2017), the older adults demonstrated super-additive effect reflected from the significant more positive-going amplitudes at the fronto-central sites around 200 ms after stimulus onset in the multisensory ([AV]) condition than the unisensory ([A] or [V]) conditions. This contrasted with the non-significant results observed in the younger comparison group. P2 was defined as the second positive peak after stimulus onset in the fronto-central sites.

### 3.3 Mean LRP amplitude

**Figure 2:**
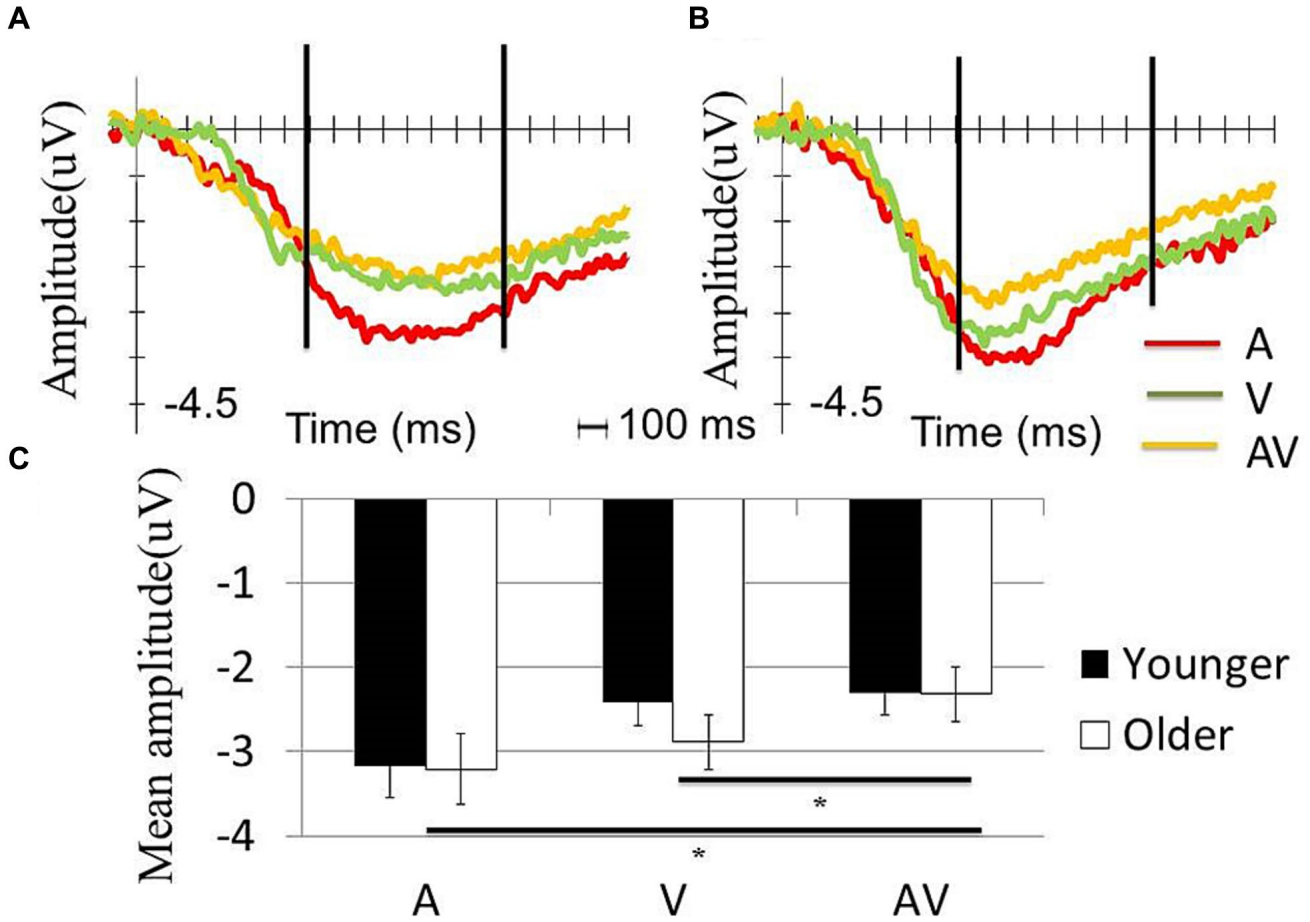
Comparison of the s-LRP waveform between the two groups. S-LRP waveform in A, V and AV conditions in **(A)** younger and **(B)** old participants. The two vertical lines in **(A,B)** showed the time window for calculating the mean amplitude of s-LRP. **(C)** Both younger and older participants showed significantly less negative going waveform in the AV condition compared with A and V condition. However, the s-LRP amplitude was comparable between the two groups within each condition.

The significance level was set as *p* < 0.05. A two-way repeated ANOVA was performed to test the effects of condition (AV, A or V) and age (older or younger) on the mean s-LRP amplitude with a time window of 700–1,500 ms. The results showed a significant condition effect (*F* (2,116) = 7.90, *p* = 0.003), but no significant age effect (*F* (1,58) = 0.21, *p* = 0.465) or interaction effect was observed (*F* (2,116) = 0.66, *p* = 0.458). A post-hoc analysis that tested the main effect of condition showed that the mean s-LRP amplitude in the [AV] condition was significantly less negative-going than that in the [A] (*t* (59) = −3.65, *p* = 0.001) or [V] condition (*t* (59) = −2.77, *p* = 0.008) (Figure 2).

**Figure 3:**
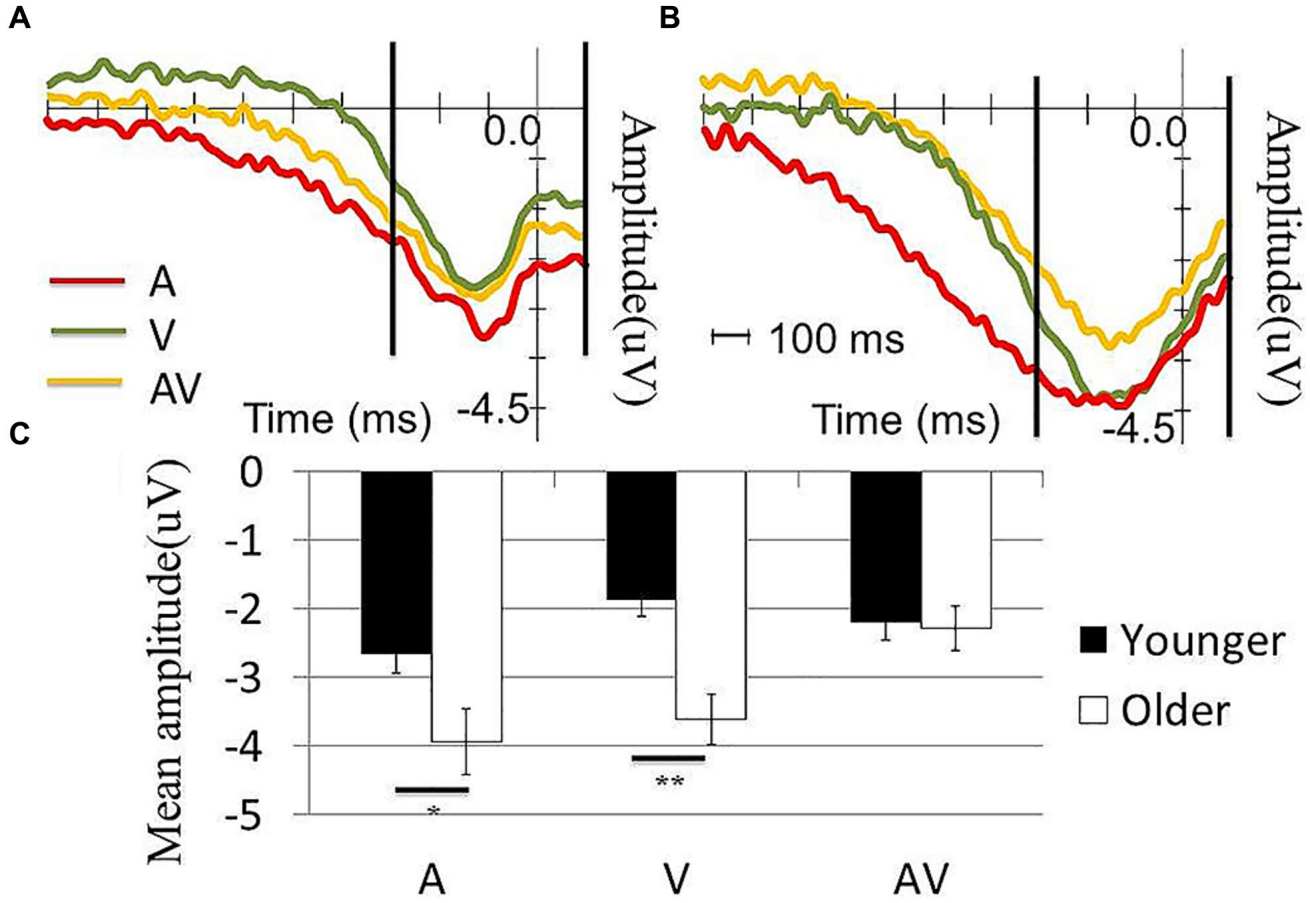
Comparison of the r-LRP waveform between the two groups. **(A)** r-LRP waveform in A, V and AV conditions in **(A)** young and **(B)** old participants. The two vertical lines in **(A,B)** showed the time window for calculating the mean amplitude of r-LRP. **(C)** The older participants showed significantly more negative going r-LRP than the younger in both A and V conditions while no significant difference was observed in AV condition.

Next, we tested the effects of condition (AV, A or V) and age (older or younger) on the mean r-LRP amplitude. The results showed a significant condition effect (*F* (2,116) = 8.73, *p* = 0.001) and age effect (*F* (1,58) = 4.39, *p* = 0.041). The interaction effect was also significant (*F* (2,116) = 4.30, *p* = 0.025). When compared with the older participants, younger participants showed significantly less negative-going mean r-LRP amplitudes in the [A] (*t* (58) = 2.27, *p* = 0.025) and [V] condition (*t* (58) = 3.92, *p* < 0.001). However, the between-group differences in the mean r-LRP amplitudes were not statistically significant (*t* (58) = 1.53, *p* = 0.131). In the younger group, no differences between the [A] and [AV] conditions (*t* (28) = −2.04, *p* = 0.051) or [V] and [AV] conditions (*t* (28) = 1.84, *p* = 0.077) were observed. In contrast, the amplitude in the [AV] condition was significantly less negative-going than the [A] (*t* (30) = −2.88, *p* = 0.007) and [V] conditions (*t* (30) = −4.82, *p* < 0.001) in the older group (Figure 3).

### 3.4 Correlation between LRP amplitude and behavioral data

**Figure 4:**
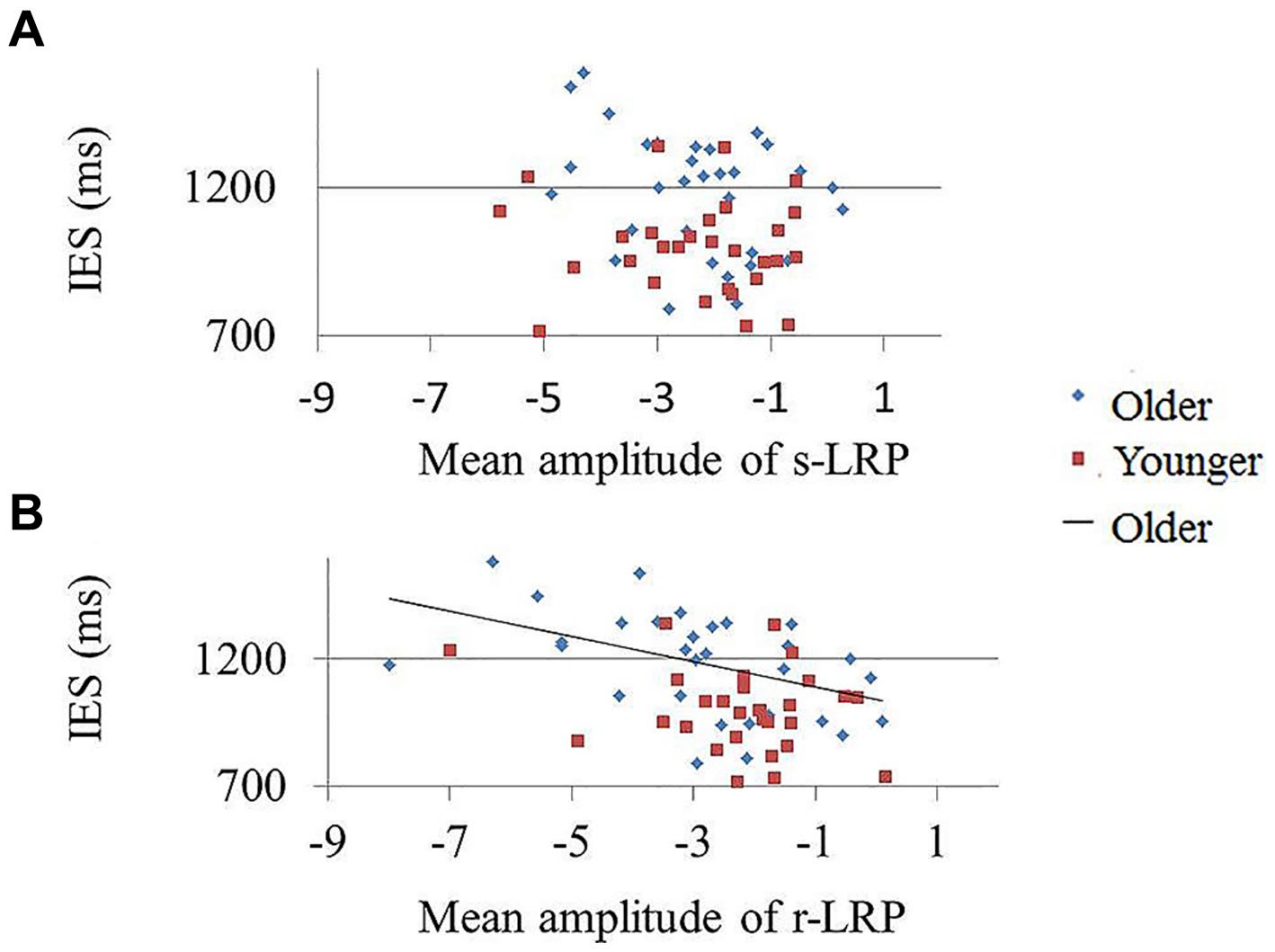
Correlation between mean r-LRP amplitude and IES in younger and older participants. The lower IES in older group was significantly correlated with the decreased amplitude of r-LRP. **(A)** The s-LRP mean amplitude and IES showed no significant correlation in both the younger and older participants. **(B)** The less negative r-LRP amplitude in AV condition was negatively correlated with less positive IES score in the older group (blue diamonds). No significant correlation between r-LRP amplitude and IES score was found in the younger participants (red squares).

The results demonstrated a non-significant correlation between the mean r-LRP amplitude and IES in the [AV] condition in the younger group (*r* = −0.23, *p* = 0.232), but a significant correlation in the older group (*r* = −0.43, *p* = 0.016) (Figure 4). No significant correlation between IES and r-LRP amplitude in unisensory conditions ([A] and [V]) were observed.

### 3.5 Regression analysis of LRP and P2 amplitudes and behavioral data

To further assess the relationship between the different ERP components and behavioral performance, a regression analysis was conducted with behavioral performance in the [AV] condition as the dependent variable, and the P2, s-LRP, and r-LRP amplitudes as the independent variables. The regression was significant only in older participants (adjusted *R*^2^ = 0.199, *F* = 3.489, *p* = 0.029), but only the r-LRP was the significant regressor (B = −67.121, *t* = −2.692, *p* = 0.012) and P2 amplitude was the marginally significant regressor (B = 16.407, *t* = 1.855, *p* = 0.075).

### 3.6 LRP onset latency

In terms of the s-LRP onset latency comparison, there was a significant interaction between condition and age (*F* (2,116) = 4.45, *p* = 0.014). The results also showed a significant main effect of condition (*F* (2,116) = 17.80, *p* < 0.001) but non-significant age effect (*F* (1,58) = 0.338, *p* = 0.715). Post-hoc analysis demonstrated that the onset latency in the [AV] condition was significantly earlier than the [A] condition in both groups (younger: 231 ms, *t* (28) = 4.083, p < 0.001; older: 76 ms, [A] & [AV]: *t* (30) = 3.244, *p* = 0.003). The onset latency in the [AV] condition was marginally earlier than the [V] condition in the younger group (64 ms, *t* (28) = 1.915, *p* = 0.066), but no significant difference was found in the older group (22 ms, *t* (30) = 1.163, *p* = 0.254).

In terms of the r-LRP onset latency, results showed significant main effects of condition (*F* (2,116) = 6.06, *p* = 0.031) and age (*F* (1,58) = 9.88, *p* = 0.003), but no significant of their interaction (*F* (2,116) = 1.44, *p* = 0.241). To examine the modulation of multisensory process on motor generation, the post-hoc comparison compared the r-LRP onset latency with the entire sample (both younger and older groups) of this study. Results showed no significant onset latency between the [AV] and [V] conditions (21 ms faster in [V]; *t* (59) = 0.56, *p* = 0.581) or between the [AV] and [A] conditions (130 ms faster in [AV]; *t* (59) = −2.18, *p* = 0.037, significance threshold of *p* = 0.017).

## 4 Discussion

Audiovisual integration enhanced motor responses in terms of faster reaction time. The faster reaction time was associated with the motor generation process modulated in the audiovisual condition. There were two important observations. Firstly, only the generation but not the preparation process contributed to the enhanced motor responses. Secondly, the enhancement and modulation effects were only significant in the older but not the younger participants. As a result, our hypotheses set for this study were partially supported.

The older participants showed significant differences in P2, s-LRP, and r-LRP in the audiovisual condition when compared with the audial and visual conditions. The results suggest that audiovisual integration was prominent in the older participants, which modulated both motor preparation and generation processes as reflected from the significant s-LRP and r-LRP, respectively. Among them, the r-LRP amplitude was a significant regressor of reaction time. The significant frontal P2 and r-LRP only observed in the older but not younger participants are new findings. More importantly, the co-existence of P2 and r-LRP together with the faster reaction times support our proposition that the super-additive effects generated from audiovisual integration would have promoted motor generation processes.

The less negative-going r-LRP amplitude revealed in older participants in the audiovisual condition reflects stronger activations in the motor cortices (Sterr and Dean, 2008). The frontal P2 is within the aging-related cognitive reserve framework (Burkhardt et al., 2022), which can be deployed by the premotor cortex (Stern et al., 2005; Quinzi et al., 2020). A less negative-going r-LRP was previously reported to associate with lower difficult level motor tasks (Nagy et al., 2020). The association of the r-LRP results with the significant frontal P2 amplitudes and faster reaction times suggest that the synchronized audiovisual inputs elicited the super-additive effect and promoted faster upper-limb motor responses among the older participants. The proposition that the P2 connects to the r-LRP further supports previous findings that the motor generation process is subserved by the cortico-thalamo-cortical route (Sherman, 2007; Cappe et al., 2012). Evidence has shown that the thalamus plays an important role in the multisensory integration (Cappe et al., 2010). The role plays by the thalamus is to relay sensory information from various modalities to the motor cortex (Cappe et al., 2009a,b). A recent study further demonstrated that sensory information relayed by the thalamus involved the multisensory vestibular region, brain stem and the motor cortex (Conrad et al., 2023).

Besides the r-LRP amplitude, the differences in the s-LRP were also significant in the audiovisual condition in the older group. The shorter latency and more negative-going s-LRP amplitude suggest that the super-additive effects resulted in a faster and stronger motor preparation process (Wild-Wall et al., 2008). However, the enhanced preparation process did not seem to have a significant impact on the reaction time produced. A previous study suggested that audiovisual effects enriched the spatial information among the response decision rules (Werner and Noppeney, 2010). Together with the non-significant between-group latency difference, our results highlight two important aspects of the r-LRP. Firstly, the specificity of the audiovisual super-additive effect to promote motor responses is likely to be via motor generation rather than preparation process. Secondly, consistent with previous studies, response preparation process is somewhat preserved during neurodegeneration (Yordanova et al., 2004; Falkenstein et al., 2006; Woods et al., 2015).

The non-significant LRP results revealed for the younger participants from the audiovisual condition were inconsistent with those reported in two previous studies (Jepma et al., 2009; Los and Van der Burg, 2013). The non-compatible audial and visual stimuli adopted by Los and Burg compared with the compatible audial and visual spatial stimuli employed in our study could have explained the inconsistency in the s-LRP results (Los and Van der Burg, 2013). The moving versus stationary hand comparisons by Jepma et al. (2009) differ from the inter-hemispheric contrasts employed in our study, which could explain the inconsistent findings in the r-LRP.

## 5 Conclusion

The audiovisual integration not only can modulate the super-additive cognitive process, but also modulate the motor related process. This study demonstrates that congruent audiovisual stimuli modulate the motor generation process for producing faster upper-limb responses in older participants. The same neural modulation effects were not observed in younger participants. The motor facilitation due to the audiovisual super-additivity effects is likely subserved by the cortico-thalamo-cortical pathway. Our proposition is supported by the significant correlations among the frontal P2, pre-motor r-LRP, and faster reaction times in the older participants. Future research should aim to understand the causal relationships between the frontal P2 and the pre-motor r-LRP as a compensatory mechanism for promoting motor functions in older adults.

## Data availability statement

The raw data supporting the conclusions of this article will be made available by the authors, without undue reservation.

## Ethics statement

The studies involving humans were approved by the Hong Kong Polytechnic University. The studies were conducted in accordance with the local legislation and institutional requirements. The participants provided their written informed consent to participate in this study. Written informed consent was obtained from the individual(s) for the publication of any potentially identifiable images or data included in this article.

## Author contributions

ZZ: Writing – original draft, Writing – review & editing, Conceptualization, Data curation, Investigation. BZ: Writing – original draft, Writing – review & editing, Data curation, Investigation, Methodology, Software. K-hT: Writing – review & editing. CW: Writing – review & editing. XH: Supervision, Writing – review & editing. CC: Supervision, Writing – review & editing.
